# Factors associated with adherence to sports and exercise among outpatients with major depressive disorder

**DOI:** 10.47626/2237-6089-2019-0109

**Published:** 2021-06-01

**Authors:** Fernanda Castro Monteiro, Felipe Barreto Schuch, Andrea Camaz Deslandes, Bruno Paz Mosqueiro, Marco Antonio Caldieraro, Marcelo Pio de Almeida Fleck

**Affiliations:** 1 Instituto de Psiquiatria Universidade Federal do Rio de Janeiro Rio de JaneiroRJ Brazil Instituto de Psiquiatria, Universidade Federal do Rio de Janeiro (UFRJ), Rio de Janeiro, RJ, Brazil.; 2 Laboratório de Neurociência do Exercício UFRJ Rio de JaneiroRJ Brazil Laboratório de Neurociência do Exercício (LaNEx), UFRJ, Rio de Janeiro, RJ, Brazil.; 3 Departamento de Métodos e Técnicas Esportivas Universidade Federal de Santa Maria Santa MariaRS Brazil Departamento de Métodos e Técnicas Esportivas, Universidade Federal de Santa Maria (UFSM), Santa Maria, RS, Brazil.; 4 Programa de Pós-Graduação em Psiquiatria e Ciências do Comportamento Universidade Federal do Rio Grande do Sul Porto AlegreRS Brazil Programa de Pós-Graduação em Psiquiatria e Ciências do Comportamento, Universidade Federal do Rio Grande do Sul (UFRGS), Porto Alegre, RS, Brazil.; 5 Departamento de Psiquiatria Hospital de Clínicas de Porto Alegre Porto AlegreRS Brazil Departamento de Psiquiatria, Hospital de Clínicas de Porto Alegre (HCPA), Porto Alegre, RS, Brazil.

**Keywords:** Adherence, major depressive disorder, mood disorder, physical activity, physical exercise, sports

## Abstract

**Introduction:**

Individuals with major depressive disorder (MDD) face more barriers to engagement in sports and exercise interventions. Evaluating clinical and demographic factors associated with adherence to sports and exercise among MDD outpatients could support development of new options and strategies to increase their participation.

**Methods:**

In a cross-sectional study, 268 depressed outpatients were evaluated (83.51% females; mean age = 50.74 [standard deviation {SD} = 10.39]). Sports and exercise participation were assessed using a question about participation frequency during the previous month. Clinical and demographic factors were evaluated. Linear regression was used to identify predictors of participation in sports and exercise.

**Results:**

MDD patients with mild symptoms of depression (odds ratio [OR] = 2.42; 95% confidence interval [95%CI] 1.00, 5.88; p = 0.04) and patients with mild to moderate symptoms (OR = 3.96; 95%CI 1.41, 11.15; p = 0.009) were more likely to engage regularly in sports and exercise than patients with more severe depression. Moreover, smoking (OR = 0.23; 95%CI 0.67, 0.80; p = 0.007) and being divorced (OR = 0.22; 95%CI 0.57, 0.86; p = 0.03) were associated with lower rates of engagement in sports and exercise.

**Conclusion:**

Our findings indicate a significant association between clinical and demographic factors and participation in sports and exercise among MDD outpatients.

## Introduction

Major depressive disorder (MDD) is one of the most common mental disorders. Lifetime prevalence is about 16% and it has a chronic course.^1^ People with severe mental illness, including MDD, have a life expectancy that is 10 to 17.5 years shorter compared to the general population.^2-4^ Researchers have consistently reported that these individuals have elevated mortality, presenting high rates of adverse health behaviors, including tobacco smoking, substance use, physical inactivity, and poor diet.^5^ About 60% of the increased mortality observed in mental illness patients is due to physical comorbidities, predominantly cardiovascular diseases.^6^ In a recent study, Firth et al. reported that individuals with mental disorders have 1.4 to 2.0 times greater risk of cardiometabolic disease than individuals without mental disorders.^7^ Moreover, individuals with depression have around 40% greater risk of developing cardiovascular disease, diabetes, metabolic syndrome, hypertension, and obesity than the general population.^7^ Along the same lines, previous studies have reported evidence of a bidirectional association between diabetes and depression,^8,9^ demonstrating that this comorbidity has a negative impact on lifestyle and quality of life, increasing healthcare costs, and susceptibility to further chronic diseases.^10,11^ Regular physical activity (PA) has been recognized as one of the most important healthy behaviors for preventing onset of many chronic diseases or reducing their severity.^7^ In people with MDD, exercise (defined as structured and systematic PA) contributes to reductions in depressive symptoms.^12^ Although people with depression can benefit from exercise, members of this subgroup are at increased risk of being sedentary and are 50% more likely to not engage in 150 minutes of moderate to vigorous PA per week.^13^ Findings from clinical samples have shown that engagement in PA is moderated by several factors, including treatment setting, since it is higher among people in outpatient care (70.9%) than in people living in a community (65.3%) or in mixed settings (17.4%).^13^ However, these findings came from a sample mostly comprising patients from high-income countries. Data from a non-clinical sample from low- and middle-income countries suggests that those who were unmarried were more likely to be physically inactive.^14^ To the best of our knowledge, there are no studies evaluating the factors associated with participation in exercise and sport with clinical samples from low- and/or middle-income countries.

Indeed, identifying demographic and clinical factors associated with exercise participation in clinical samples can support development of successful interventions in these individuals, especially in low- and middle-income countries. Given the shortcomings in currently available evidence, the aims of the present study were to 1) evaluate the frequency of participation in sports and exercise among outpatients with MDD from a middle-income country and 2) explore the demographic and clinical factors associated with participation in sports and exercise among these patients.

## Methods

This is a cross-sectional study including outpatients with a clinical diagnosis of MDD confirmed by the MINI^15^ who started treatment between 2005 to 2015 at the Mood Disorders Treatment Program (PROTHUM) run by the Hospital de Clínicas de Porto Alegre (HCPA), in the city of Porto Alegre, southern Brazil. The study analyzed data on these patients extracted from a database. Our inclusion criteria were patients from 18 to 65 years old; diagnosed with MDD in accordance with the Mini-International Neuropsychiatric Interview (MINI-Plus)^15^ and scoring 8 points or more in the HAM-D scale; who had been admitted for outpatient care at PROTHUM, HCPA. Our exclusion criteria were patients diagnosed with bipolar disorder, schizophrenia, schizoaffective disorder, and patients with a primary diagnosis of Substance Use Disorder. Written informed consent was obtained from all participants when they started treatment at PROTHUM. The procedure was approved by the research ethics committee at the HCPA (protocol no. 2016-0540).

Demographic variables were categorized as follows: age (18 to 35; 35 to 50; 51 to 65; and over 65 years old; [we extracted and established these cut-off points at ages that yielded groups with similar numbers of individuals]); sex (male or female); body mass index (BMI) (≤ 25; 25-30; 31-35; and > 35); skin color (white; black; indigenous; Asian; mestizo); marital status (single; married; divorced; widowed); educational level in years (≤ 8; 9 to 12; and > 13); economic activity (has income; no income; retired; and social security benefits); and socioeconomic status (Pooled A+B; C; D+E) according to the Brazilian Association of Research Companies (ABEP) classification. The ABEP is an agency responsible for managing research quality and guidelines for adoption of the New Brazilian Economic Classification Criteria.^16^

We used the following assessment instruments:

1. Mini-International Neuropsychiatric Interview. The MINI-Plus is administered by trained health professionals. It contains 17 sections to diagnose current or past episodes of psychotic disorders, mood disorders, anxiety disorders, and alcohol and drug use disorders.^17^ It also includes a section to classify a Major Depressive Episode as melancholic or non-melancholic according to the DSM criteria.
2. Hamilton Depression Rating Scale (HAM-D).^18^
3. Beck Depression Inventory (BDI).^19^
4. Sports and Exercise Participation. Sports and exercise participation was assessed using a single question: “On average, how many hours per week did you spend participating in sports or exercise during the last month?” There were five response options: none; one hour or less; between one and two hours; between two and four hours; and more than four hours per week. The responses none and less than one hour were categorized as physically inactive and all other responses were categorized as physically active.
5. The Core Assessment of Psychomotor Change.^20-23^
6. Global Assessment Functioning (GAF).^24^
7. The World Health Organization Quality of Life Questionnaire-Brief Version (Whoqol-BREF).^25,26^ In the present study, the participants were classified into tertiles by their quality-of-life scores.
8. Clinical Global Impression (CGI).^27^

Other independent variables were collected on clinical variables, such as previous hospitalization (yes; no); smoking (yes; no); menopause (yes; no); medication use (yes; no); and the type of medication used. In the statistical analysis, descriptive analyses were conducted using means and standard deviations for continuous measures and frequencies for categorical variables. Candidate correlates of PA, sports, and exercise were selected based on the literature.

We conducted multivariable logistic regression analyses. First, we assessed the sociodemographic correlates of PA by constructing a model that included all the sociodemographic variables (age, sex, education, income, marital status, skin color, BMI, socioeconomic status, and economic activity). Next, we analyzed the associations between each of the other clinical correlates and physical inactivity, while adjusting for all the sociodemographic variables mentioned above. All variables were included in the models as categorical variables, except for age and continuous variables. Results from the regression analyses are presented as odds ratios (ORs) together with their 95% confidence intervals (95%CIs). The level of statistical significance was set at p < 0.05. We used the Statistical Package for the Social Sciences (SPSS) statistical program.

## Results

The final sample consisted of 268 individuals in outpatient care (83.51% females; mean age = 50.74 [standard deviation {SD} = 10.39]). On average, they were overweight (mean BMI = 28.37; SD = 6.42), predominantly white (74.6%), and living with a partner (57%), had spent 7.27 (SD = 3.65) years in education, and were members of socioeconomic status class C (52.3%). Most participants engaged in none or less than one hour of sport and exercise, which categorized them as physically inactive (84.9%). The full details of the sample can be observed in Table 1.

**Table 1:** Participants’ characteristics

Variable	
Age (years), mean (SD)	50.74 (10.39)
Sex (female)	233 (83.51)
Body mass index, mean (SD)	28.37 (6.42)
Skin color	
White	208 (74.6)
Black	58 (20.8)
Indigenous, mestizo, Asian	2 (0.7)
Marital status	
Living with a partner/married	159 (57)
Single	20 (7.2)
Widowed	26 (9.3)
Divorced	67 (24)
Years of study (years), mean (SD)	7.27 (3.650)
Socioeconomic status	
A	2 (0.8)
B	63 (24)
C	137 (52.3)
D	56 (21.4)
E	4 (1.5)
Smoker (yes)	190 (72)
BDI (score), mean (SD)	34.78 (9.91)
HAM-D (score), mean (SD)	21.5 (5.59)
Melancholic (CORE) (yes)	70 (26.5)
Medications (yes)	
SSRI	192 (72.5)
Tricyclics	128 (48.5)
MAOI	1 (0.4)
Lithium	39 (14.8)
Antipsychotics	73 (27.7)
Benzodiazepines	45 (29)
Quality of life (score), mean (SD)	
Psychological	30.35 (14.56)
Physical	29.41 (13.30)
Social relationships	42.08 (21.74)
Environment	43.33 (12.40)

Data presented as n (%), unless otherwise specified.

BDI = Beck Depression Inventory; CORE = The Core Assessment of Psychomotor Change; HAM-D = Hamilton Depression Rating Scale; MAOI = monoamine oxidase inhibitors; SD = standard deviation; SSRI = selective serotonin reuptake inhibitors.

Of the demographic variables, being divorced was associated with a lower rate of participation in sport and exercise (OR = 0.22; 95%CI 0.57 to 0.86; p = 0.03). We did not find any significant associations with the other demographic variables (Table 2).

**Table 2:** Demographic variables and associations with participation in exercise and sports among outpatients with major depressive disorder

Variable/Category	OR	95%CI lower	95%CI upper	p
Age				
18-35	0.58	0.11	3.04	0.52
35-50	0.36	0.16	1.95	0.36
51-65	0.63	0.18	2.12	0.45
> 65*	1	1	1	1
Sex				
Men × women	1.80	0.81	3.99	0.14
BMI				
≤ 25	1.26	0.42	3.80	0.67
25-30	1.98	0.67	5.83	0.21
31-35	1.17	0.33	4.15	0.80
> 35*	1	1	1	1
Skin color				
White × black/indigenous/Asian/mestizo	2.27	0.85	6.08	0.10
Marital status				
Single	0.58	0.12	2.67	0.48
Living with a partner/married	0.78	0.28	2.10	0.62
Divorced	0.22	0.57	0.86	**0.03**^**†**^
Widowed*	1	1	1	1
Years of study				
≤ 8	0.66	0.20	2.16	0.49
9-12	0.64	0.18	2.30	0.50
> 13*	1	1	1	1
Socioeconomic status				
A+B	1.15	0.56	4.20	0.39
C	1.47	0.59	3.65	0.40
D+E*	1	1	1	1
Economic activity				
Has income	0.69	0.19	2.42	0.57
No income	1.39	0.56	3.49	0.46
Retired	1.51	0.56	4.07	0.41
Social security benefit*	1	1	1	1

95%CI = 95% confidence interval; BMI = body mass index; OR = odds ratio.

* Reference group.

^**†**^ Correlation is significant at the 0.05 level.

With regard to clinical variables, patients with mild symptoms of depression according to the HAM-D (OR = 2.42; 95%CI 1.00 to 5.88; p = 0.04) and mild to moderate according to the BDI (depression severity = light to moderate) were more likely to participate in sport and exercise than patients with more severe depression (OR = 3.96; 95%CI 1.41 to 11.15; p = 0.009). Patients with melancholic symptoms according to MINI-Plus were less likely to engage in sport and exercise (OR = 0.36; 95%CI 0.17 to 0.18; p = 0.003) (Table 3). Participants with lower scores in the psychological domain of the WHOQOL-BREF (OR = 0.42; 95%CI 0.19 to 0.91; p = 0.03) and lower GAF scores (OR = 0.42; 95%CI 0.17 to 0.99; p = 0.04) were less likely to engage in regular sports and exercise (Table 3). Smoking (OR = 0.23; 95%CI 0.67, 0.80; p = 0.007) and being divorced (OR = 0.22; 95%CI 0.57, 0.86; p = 0.03) were associated with lower engagement in sports and exercise (Table 3). Lastly, participants with higher scores in environmental quality of life domain of the WHOQOL-BREF (OR = 0.44; 95%CI 0.18 to 1.02; p = 0.05) were more likely to engage in sports and exercise (Table 3).

**Table 3:** Clinical factors associated with participation in exercise and sports among outpatients with major depressive disorder

Variable/Category	OR	95%CI lower	95%CI upper	p
Depression severity (HAM-D)				
Mild	2.42	1.00	5.88	0.04*
Moderate	1.55	0.68	3.51	0.254
Severe^†^	1	1	1	1
Depression severity (BDI)				
Mild to moderate	3.96	1.41	11.15	0.009*
Moderate to severe	0.92	0.47	2.28	0.92
Severe^†^	1	1	1	1
Melancholia (MINI)				
Yes × no	0.36	0.17	0.18	0.003*
Melancholia (CORE)				
Yes × no	0.59	0.26	1.34	0.21
Suicide risk				
No/mild × moderate/high	1.77	0.89	3.49	0.10
Suicide attempt				
Yes × no	1.14	0.58	2.26	0.68
Previous hospitalization				
Yes × no	1.11	0.52	2.36	0.77
Smoking				
Yes × no	0.23	0.67	0.80	0.007*
Menopause				
Yes × no	1.82	0.80	4.13	0.15
SSRI use				
Yes × no	0.68	0.34	1.36	0.28
Tricyclics				
Yes × no	1.60	0.82	3.11	0.16
Lithium				
Yes × no	0.55	0.18	1.63	0.28
Antipsychotics				
Yes × no	0.48	0.20	1.13	0.94
Benzodiazepines				
Yes × no	0.99	0.36	2.75	0.99
Clinical global impression				
Higher × lower	0.81	0.24	3.03	0.81
GAF				
Tertile 1	0.42	0.17	0.99	0.04*
Tertile 2	0.82	0.38	1.75	0.61
Tertile 3^†^	1	1	1	1
Physical domain				
Tertile 1	0.49	0.22	1.08	0.08
Tertile 2	1.47	0.61	3.54	0.38
Tertile 3^†^	1	1	1	1
Psychological domain				
Tertile 1	0.42	0.19	0.91	0.03*
Tertile 2	0.91	0.37	2.19	0.83
Tertile 3^†^	1	1	1	1
Social relationships domain				
Tertile 1	0.69	0.29	1.64	0.41
Tertile 2	0.71	0.33	1.51	0.37
Tertile 3^†^	1	1	1	1
Environment domain				
Tertile 1	0.44	0.18	1.02	0.05*
Tertile 2	0.67	0.29	1.52	0.34
Tertile 3^†^	1	1	1	1

95%CI = 95% confidence interval; BDI = Beck Depression Inventory; CORE = The Core Assessment of Psychomotor Change; GAF = Global Assessment of Functioning; HAM-D = Hamilton Depression Rating Scale; MINI = Mini-International Neuropsychiatric Interview; OR = odds Ratio; SSRI = selective serotonin reuptake inhibitors.

* Correlation is significant at the 0.05 level.

^†^ Reference group.

## Discussion

We investigated factors associated with participation in sports and exercise among outpatients with MDD from a middle-income country. Participants with mild depressive symptoms and with non-melancholic episodes were more likely to be physically active. Also, smokers, divorcees, and patients with poorer functionality and lower psychological quality of life were less likely to engage in exercise. Firth et al. found that around 30% of patients with MDD had alcohol use disorder; in addition, these patients are more likely to smoke and be dependent on nicotine, are less likely to quit, and are more likely to relapse. These authors also found that around 60-70% of MDD patients did not meet PA guidelines, being sedentary for 8.5 hours per day.^7^

Our findings indicate that clinical and demographic factors might moderate participation in exercise and sports. For example, marital status, severity of symptoms, the subtype of depression, global functioning, and quality of life seem to moderate exercise participation. Knowledge about correlates of PA habits helps to identify high-risk individuals who are less likely to engage in physical exercise and who may therefore require intensified and targeted interventions.^28^ For example, a study investigating the impact of anxiety disorders on the risk of low PA levels in outpatients with a lifetime diagnosis of panic disorder showed, for the first time, that cognitive symptoms of anxiety were not significant predictors of low PA, but that individual somatic symptoms of anxiety may better predict low levels of PA.^29^

Additionally, our findings indicate that melancholic patients found it more difficult to get involved in sports and exercise. There is evidence in the literature showing that symptoms considered important to diagnosing melancholia in depressed patients include mood nonreactivity and psychomotor retardation.^30^ It is therefore possible that in these patients it is necessary to achieve an initial improvement with other interventions to promote exercise before or in conjunction with an intervention. It is also important that future studies investigate how practices that involve less activation or intensity may be a better choice in this population with a phenotypic presentation - described by more anergic characteristics. Also, smoking affects lung capacity and might be associated with reduced exercise capacity, increasing the perception of effort even at lighter intensities. Thus, in this sense, interventions to reduce tobacco consumption can also help to promote physical exercise and sports.

We found that patients with mild symptoms of depression (or mild to moderate symptoms) were more likely to be regularly engaged in sports and exercise, while those with severe symptoms, smokers, and divorcees exhibited lower levels of engagement in sports and exercise. Individuals who do not meet PA recommendations experience a negative impact on mental health and quality of life.^31^ Besides this, Vancampfort et al.^28^ found that loneliness, less social connectivity, and lack of social support are underlying reasons explaining why lower levels of social relationships are associated with higher levels of sedentary behavior^32^ and depression.^33^ In another study, the authors found that adding exercise to the conventional treatment for severely depressed inpatients was an effective strategy for reducing depressive symptoms and improving quality of life in the physical and psychological domains.^34^ Moreover, it is necessary to understand the history of PA and promote feasible interventions to reduce physical inactivity in order to lower rates of many different types of disease and improve overall health.^35^

Finally, given the complexity of factors involved in participation in exercise and sport among patients with MDD and the high rates of physical inactivity observed, it could be helpful to provide support through multidisciplinary teams, including psychiatrists, psychologists, and other health professionals, to engage this population in sports and exercise programs, raising awareness of the importance of being physically active.

One limitation of the present study is the cross-sectional nature of assessment of sports and exercise measures. As a result, cause and effect relationships cannot be deduced with certainty. Second, participation in sports and exercise was measured with a single question, rather than with a specific questionnaire about PA. Strengths include the fact that our study used a structured psychiatric diagnosis assessment in a clinical sample, considering the heterogeneity of depressive symptoms. A second strength is the significant sample size from a middle-income country.

## Conclusion

This study demonstrates that several demographic and clinical factors are associated with physical inactivity in MDD outpatients in middle-income countries (e.g., Brazil). According to these findings, evaluation of clinical and demographic correlates could aid development of better strategies for increasing sports and exercise levels in depressed subjects, helping to decrease depressive symptoms and improve health.
